# Supplementary material for: Scalable and switchable CO2-responsive membranes with high wettability for separation of various oil/water systems
Source: Nat Commun. 2023 Feb 27;14:1108. doi: 10.1038/s41467-023-36685-9 (PMC9970982; doi:10.1038/s41467-023-36685-9)
Supplement: Supplementary file 1 — Supplementary Information [file 41467_2023_36685_MOESM1_ESM.pdf]

**Scalable and switchable $CO_2$-responsive membranes with high wettability for separation of various oil/water systems**

Yangyang Wang,[1¦] Shaokang Yang,[2¦] Jingwei Zhang,[3¦] Zhuo Chen,[3] Bo Zhu,[4] Jian Li,[5] Shijing Liang,[6] Yunxiang Bai,[1] Jianhong Xu,[3] Dewei Rao,[2] Liangliang Dong*[1], Chunfang Zhang[1], Xiaowei Yang[7]

1 Key Laboratory of Synthetic and Biological Colloids, Ministry of Education, School of Chemical and Material Engineering, Jiangnan University, Wuxi 214122, P. R. China.

2 School of Materials Science and Engineering, Jiangsu University, Zhenjiang 212013, P. R. China.

3 The State Key Laboratory of Chemical Engineering, Department of Chemical Engineering, Tsinghua University, Beijing 100084, P. R. China.

4 Key Laboratory of Eco-textiles, Ministry of Education, Jiangnan University, Wuxi 214122, P. R. China.

5 Laboratory of Environmental Biotechnology, Jiangsu Engineering Laboratory for Biomass Energy and Carbon Reduction Technology, Jiangsu Key Laboratory of Anaerobic Biotechnology, School of Environmental and Civil Engineering, Jiangnan University, Wuxi 214122, P. R. China.

6 National Engineering Research Center of Chemical Fertilizer Catalyst, Fuzhou University, Fuzhou 350002, P. R. China.

7 School of Chemistry and Chemical Engineering, Shanghai Jiao Tong University, Shanghai, 200240, P. R. China.

Corresponding author: liangliangdong@jiangnan.edu.cn.

¦ These authors contribute equally to this work.

## 1. Materials and methods

### 1.1. Materials

Polyester fabric was kindly provided from Dr. Bo Zhu (School of Textiles Science and Clothing, Jiangnan University). Tetrahydrofuran (THF), hexane, methylbenzene, isooctane and methyl methacrylate (MMA) were obtained from Beijing Chemical Co., Ltd (Chemical purity). Soybean oil was purchased from Macklin Biochemical Co., Ltd (Shanghai, Chian). Silicone oil was purchased from Zhonglan Chenguang Chemical Research and Design Institute Co., Ltd (Sichuan, China). Bovine serum albumin (BSA) was purchased from Aladdin Biochemical Technology Co., Ltd (Shanghai, China). Tetracycline (TC) was purchased from Beijing Innochem Science & Technology co., Ltd (Beijing, China). Span80 and lauryl sodium sulfate (SDS) were purchased from Sinopharm Chemical Reagent Co., Ltd (Shanghai, China). N, N-diethylaminoethylmethacrylate (DEAEMA) was obtained from Tokyo Chemical Industry Co. Ltd (Japan). Azodiisobutyronitrile (AIBN, Beijing Chemical Technology Co., 98%) was recrystallized in $CHCl_3$ twice before use. Nitrogen (99.99%) and carbon dioxide (99.99%) were supplied by Wuxi Xinnan Chemical Gas Co. Ltd. (China). The water used in all experiments was deionized water. All chemicals were used as received without further purification.

### 1.2. Methods

*1.2.1. Synthesis of PMMA-co-PDEAEMA copolymer*

The PMMA-co-PDEAEMA copolymer was synthesized by radical polymerization as shown in Supplementary Fig. 1. The typical procedure is as follows: MMA and DEAEMA monomers in a certain molar ratio as well as AIBN were added into a round-bottom flask and then dissolved in THF by stirring at room temperature to form a uniform solution. Next, the solution was degassed by three freeze/vacuum/nitrogen cycles and then put into an oil bath at 65 °C under magnetic stirring. After co-polymerization reaction proceeded for 48 h, the purified copolymer was isolated by hexane precipitation after dissolving in THF. Finally, the obtained product was dried under vacuum.

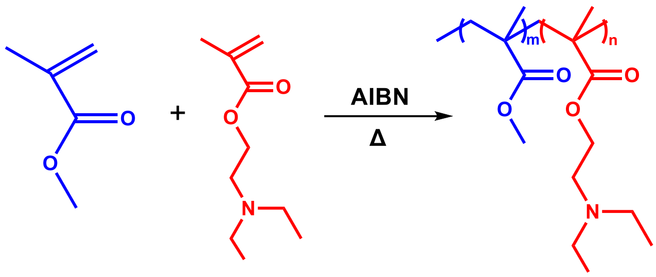


**Supplementary Figure 1.** Synthesis route of PMMA-co-PDEAEMA copolymer

*1.2.2. Fabrication of PPFMs*

The typical fabrication process of PPFMs through CFCS method is as follows: 10 wt % of PMMA-co-PDEAEMA copolymer in THF solution was filtered through a 0.45 mm PTFE filter to remove insoluble impurities. Next, a piece of polyester fabric was fixed inside of microporous gap formed by two pieces of superimposed acrylic plates. Subsequently, the resulting polymer solution was slowly injected the edge of the gap and then gradually covered the surface of fabric under the capillary force at room temperature. Finally, the as-prepared PPFMs were dried in a vacuum oven at 50 °C for 24 h.

**2. Characterization methods**

The chemical structures of copolymers were characterized by a FTLA 2000 type Fourier transform infrared (FT-IR) spectrometer with scan range of 4000-400 $cm^{-1}$ and $^1H$ NMR (BRUKER400, Germany). Molecular weight and molecular weight distribution were measured with gel permeation chromatography (GPC, Waters GPC System) at 35 °C using polystyrene standards an THF as the eluent. The chemical structures of membranes were recorded by the infrared spectra using a Thermo Nicolet 6700 spectrometer equipped with a germanium attenuated total reflection (ATR) accessory at a resolution of 4 $cm^{-1}$. The morphologies of membranes were investigated by Hitachi S4800 scanning electron microscope (SEM) with an EDAX system (Octane Super) at an accelerating voltage of 3 kV. The copolymer coating thickness of membranes was measured by the EDS line scan. Since the PMMA-PDMAEMA copolymer has similar C, O and H elements to fabric substrate, it is difficult to directly measure the coating thickness based on these elements. In order to solve this problem, we added 50 mg/mL $FeCl_2$ as tracer agent into polymer solution during the membrane preparation. Because the Fe element can be easily detected through EDS line scan, the coating thickness can be measured based on the distribution of Fe element along the cross section of membranes. The elemental composition of the membrane surface was

characterized by X-ray photoelectron spectrometer (XPS) (Kratos, Axis supra) equipped with an Al/Ka monochromatic source at energy of 1253.6 eV. Contact angle measurement was performed using a Contact Angle Measurement Device (Dataphysics OCAH 200, German). In situ FT-IR measurements were performed using a Nicolet 6700 spectrometer equipped with a reaction cell, which were obtained over a range of 4000 to 650 $cm^{-1}$ by averaging 512 scans at a resolution of 4 $cm^{-1}$. The mechanical properties of the membranes were studied at 25 °C using an WCT-10 electronic universal testing machine (Shenzhen, China) equipped with a 50 N load cell. The underwater adhesion forces of oil droplet (lubricating oil) on the PPFMs were measured using a high-sensitivity microelectromechanical balance system (K100, KRUSS, Germany). Optical microscope images of the original emulsions and filtrates were captured via an ultra-depth three-dimensional microscope (VHX-1000C, KEYENCE, Japan). The droplet size distribution was obtained by a Malvern Zeta sizer Nano ZS instrument. The pore size and the corresponding pore size distribution of the PPFMs was measured using a pore-size distribution analyzer (PMI, CFP-1500AEX) with a test area of 3.14 $cm^2$. The roughness of membrane surface was analyzed by a 3D laser microscopic imaging system (VK-150K, KEYENCE, Japan).

**3. COMSOL simulation**

A multiphase CFD simulation with level set method was conducted by COMSOL Multiphysics 5.6 (COMSOL Inc.) to get the velocity fields and pressure fields[1]. Multi-gird was used to assist the solving process due to the large gap between the feature size and the main size. The geometric model in this simulation consisted of two cubes. The former represented the gap between the substrate and the plate (1100 μm×1100 μm×h), and the latter represented the substrate that did not allow fluid to flow (1000 μm×1000 μm×135 μm). The center point of the whole system was the original point, and the direction of liquid flow was the positive direction of the X axis, the Z axis was perpendicular to the substrate (Gravity is negative along the z axis.), and the positive Y direction was determined by the right-hand coordinate principle. The grid density was set at normal mode.

The control equations for the system were N-S equations:

$$\rho \frac{\mathrm{D}u}{\mathrm{D}t} = \rho F_{\mathrm{M}} - \nabla p + \mu \nabla^2 u + F_s \tag{S1}$$

$$\rho \nabla \cdot u = 0 \tag{S2}$$

In the equations, u means the velocity, t means time, ρ means the density of the fluid,

FM means mass force, p means pressure, μ means viscosity of the fluid, and Fs means the interfacial tension[2]. The interfacial tension in the LSM was defined by the following equation:

$$F_s = -\frac{\gamma\delta}{\varepsilon}\nabla\cdot(\nabla\phi)\nabla\phi \tag{S3}$$

In this equation, ε means thickness parameter (set as default), γ means the interfacial tension, δ is a Dirac function which defines the surfaces between the gas and liquid:

$$\delta = \begin{cases} 1 & |\phi| \le \varepsilon \\ 0 & |\phi| \ge \varepsilon \end{cases} \tag{S4}$$

The level set function evolution equation used in the simulation was:

$$\frac{\partial\phi}{\partial t} + u\cdot\nabla\phi = \lambda\nabla\cdot\left(\varepsilon\nabla\phi - \phi(1-\phi)\frac{\nabla\phi}{|\nabla\phi|}\right) \tag{S5}$$

In this equation, λ is the reinitializing parameter (set as default) of the LSM.

For the boundary and initial conditions, the settings were as following. In order to simulate the situation in the experiment, the inlet velocity was chosen as a step function (the unit was m/s).

$$u(t) = \begin{cases} 2\times10^{-5} (0 < t < 0.2) \\ 0 \quad (t > 0.2) \end{cases} \tag{S6}$$

In order to ensure the solvability of the equations, a smooth option with continuous second derivatives were chosen. The entrance was the entire side of the rectangle. Outlet was the entire rectangle opposite the inlet. Outlet boundary condition was chosen as atmospheric pressure.

No penetration and no slip boundary conditions were selected at interfaces other than entrances and exits. The initial conditions for all systems were chosen as v = 0 m/s and p = 0 Pa at t = 0 s. The initial level set function in the system was set to be 0 at t = 0 s, which represented air in the system.

**4. DFT simulation**

DFT simulations with a plane-wave basis set were performed using Vienna Ab initio Simulation Package (VASP) program[3]. The electron exchange correlation was described by the generalized gradient approximation (GGA) with the function of Perdew-Burke-Ernzerhof[4]. In order to describe the van der Waals interactions between substrate (DEAEMA) and adsorbed species, DFT-D3 empirical correction of Grimme was carried out[5]. Fully relaxed geometric structures were simulated with 20 × 20 × 10 cells which is big enough to avoid the interactions between periodic images. The ion-

electron interactions were described by the projector augmented wave (PAW) method, with the plane-wave kinetic energy cutoff of 500 eV[6]. The force and energy convergence criterion were set to be $10^{-2}$ eV/Å and $10^{-5}$ eV, respectively. The Brillouin zone integration in k space was performed on a 3 × 1 × 3 k-point mesh sampled using the Monkhorst-Pack scheme[7]. The climbing nudged elastic band (CI-NEB) method was adopted to investigate the protonation mode of DEAEMA and the results were used to draw moving pictures. An explicit water layer was not considered in this work due to the computational cost whereas an implicit solvation model with a dielectric constant of 80 was employed to describe a water solvation model[8].

After the geometric structure optimization by VASP, CP2K (an open source quantum chemistry and solid state physics software package) and Multiwfn 3.6 programs (an extremely powerful program for realizing electronic wavefunction analysis) were combined for visualization of weak intermolecular interactions[9]. Meanwhile, Independent Gradient Model analysis based on Hirshfeld (IGMH) was also performed to visualize the weak interaction between the substrate and adsorbed species, in which the substrate and adsorbed species were treated as two parts[10]. The maps of the weak interactions between individual fragments were drawn with VMD software[11]. Scatter diagrams based on δg versus $\mathrm{sign}(\lambda_2)\rho$ are drawn to verify the strength of the weak interaction, where δg is found to be able to reasonably reveal inter-fragment interactions. $\mathrm{Sign}(\lambda_2)\rho$ denotes product of electron density, ρ, and sign of the second largest eigenvalue of Hessian matrix of ρ, $\mathrm{sign}(\lambda_2)$. ρ in interaction region is generally positively correlated to interaction strength, while $\mathrm{sign}(\lambda_2)$ has certain capacity of distinguishing whether the interaction is attractive or repulsive.

## 5. Separation performance of PPFMs

### *4.1. Immiscible Oil/Water Separation Experiments*

A piece of PPFM was sealed between two Teflon fixtures which were connected to two glass funnels with an effective separation area of 3.14 $cm^2$. The freshly prepared mixtures composed of water and light oil were poured onto the membrane, respectively. The driving force of the separation process solely came from its own gravity. The oil phase or water phase would be separated effectively by switching the $CO_2/N_2$ bubbling. Then the oil/water separation efficiency was measured for three times in one separation cycle, being calculated by the following equation:

$$R = (C_f - C_p)/C_f \times 100\% \quad \text{(S7)}$$

where $R$ is the separation efficiency, $C_f$ and $C_p$ represent the oil or water content in the

original oil/water mixture and collected filtrate and, respectively.

The permeate flux of the PPFMs was calculated by the following equation:

$$J = V/(At) \qquad (S8)$$

where $J$ is the permeate flux of membrane, $V$ is the volume of oil or water that permeates through the membranes; $A$ is the effective area of the membranes, and t is the filtration time.

*4.2. Emulsion Separation Experiments*

The different stabilized O/W and W/O emulsions were prepared in terms of two types of surfactants including SDS and Span 80, respectively. It is worth noting that five kinds of oils were used for preparing each type of emulsions including hexane, toluene, isooctane, soybean oil and silicone oil. Then the surfactant-stabilized O/W emulsions were prepared by dropwise adding different oils into deionized water in a volume ratio of 1/99 with the addition of 1 mg $mL^{-1}$ of SDS. For the surfactant-stabilized W/O emulsions, water and different oils were mixed in a volume ratio of 1/99 with the addition of 6 mg $mL^{-1}$ of Span 80. Finally, all aforementioned emulsions were intensively stirred for at least 24 h before use. Subsequently, the controlled separation process of all ten kinds of emulsions was achieved under the gravity by reversibly bubbling $CO_2/N_2$. The separation efficiency and permeate flux were calculated according to equation (S7) and equation (S8) above.

*4.3. Self-cleaning Performance experiments*

To evaluate the self-cleaning property of the membranes, the water-in-hexane emulsion, the hexane-in-water emulsion with or without BSA protein and TC, were used as contamination systems, respectively. The variation of permeating flux and separation efficiency of the membranes with respect to filtration time during every cycle of emulsion separation was recorded. After filtration of the emulsion, the membrane was immersed in water with bubbling $CO_2$ for 15 min and the next cycle of filtration was then conducted.

## 5. Supplementary figures and tables

Supplementary Table 1. Properties of the obtained PMMA-co-PDEAEMA copolymer

| MMA: DEAEMA[a] | MMA: DEAEMA[b] | MMA: DEAEMA[c] | Mn (g/mol) | Mw (g/mol) | PDI |
|---|---|---|---|---|---|
| 0.5 : 1 | 0.92 : 1 | 0.94 : 1 | 17800 | 40600 | 2.3 |
| 1 : 1 | 1.85 : 1 | 1.88 : 1 | 18400 | 44000 | 2.4 |
| 1.5 : 1 | 2.78 : 1 | 2.74 : 1 | 19700 | 48900 | 2.4 |
| 2 : 1 | 3.70 : 1 | 3.68 : 1 | 26700 | 58200 | 2.2 |

[a] Mass ratio of MMA and DEAEMA; [b] Molar ratio of MMA and DEAEMA; [b] Molar ratio of MMA and DEAEMA calculated from H[1] NMR

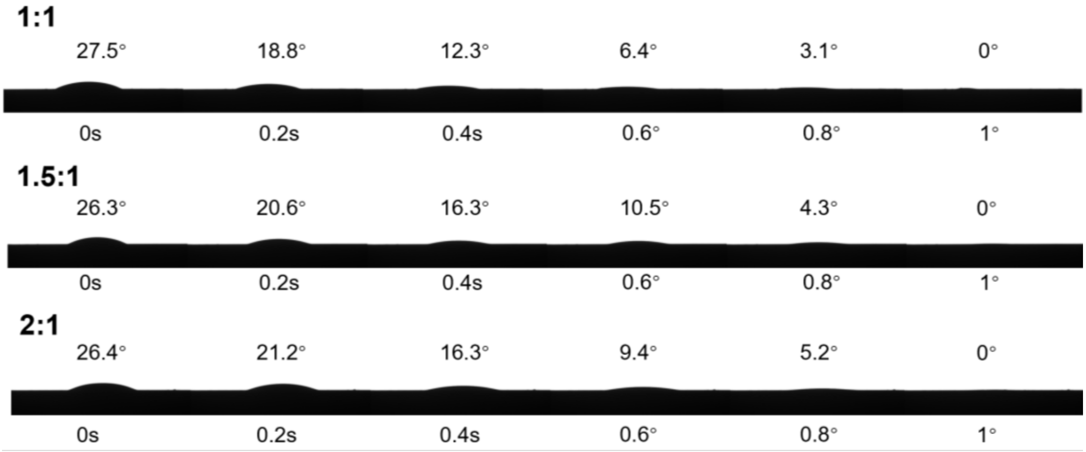


**Supplementary Figure 2.** Dynamic CA transformation of PMMA-*co*-PDEAEMA copolymer droplet with different MMA/DEAEMA ratios on the surface of the acrylic plate over time.

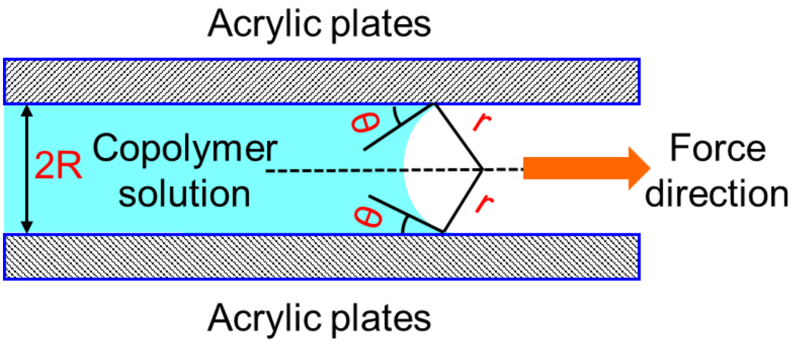


**Supplementary Figure 3.** Cross-section schematic of the rinse liquid (as shown in light blue) meniscus formed between two superimposed acrylic plates.

Capillary force is a consequence of the formation of a meniscus in the liquid between two superimposed acrylic plates. The magnitude of the capillary force can be calculated based on the Laplace equation[12], which is described as follows:

$$\Delta P = \frac{2\gamma}{r} = \frac{2\gamma \cos\theta}{R} \quad \text{(S9)}$$

where ΔP is the pressure difference, γ is the surface tension of the liquid, θ is the angle between solid-liquid interface and liquid-gas interface, R and r are capillary radius and radius of curvature respectively. Based on the equation (S9), it can be found that the diffusion force of solution in capillary is inversely proportional to the contact angle (θ) and capillary radius (R). That is, the smaller contact angle (θ) and capillary radius (R) (that is, gap width in this work) is more favorable to the diffusion of the solution in the micropores.

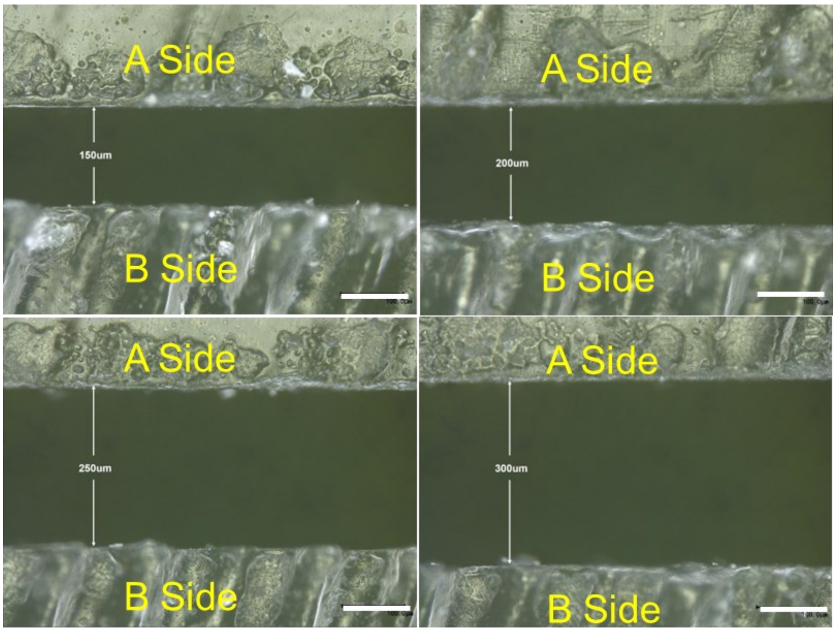


**Supplementary Figure 4.** Optical images of two pieces of superimposed acrylic plates with different gap width. The scale bar is 100 μm.

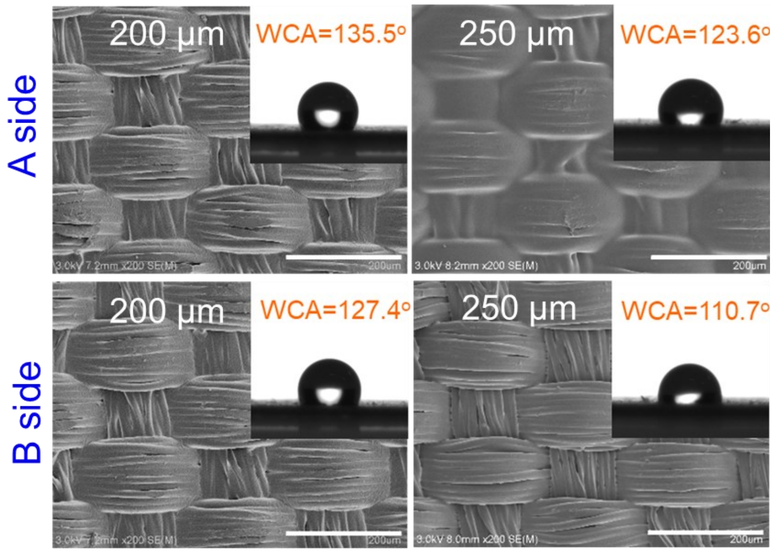


**Supplementary Figure 5.** SEM images and WCA of as-prepared PPFM (MMA/DEAEMA ratio of 0.5) surfaces on two sides. The scale bar is 200 μm.

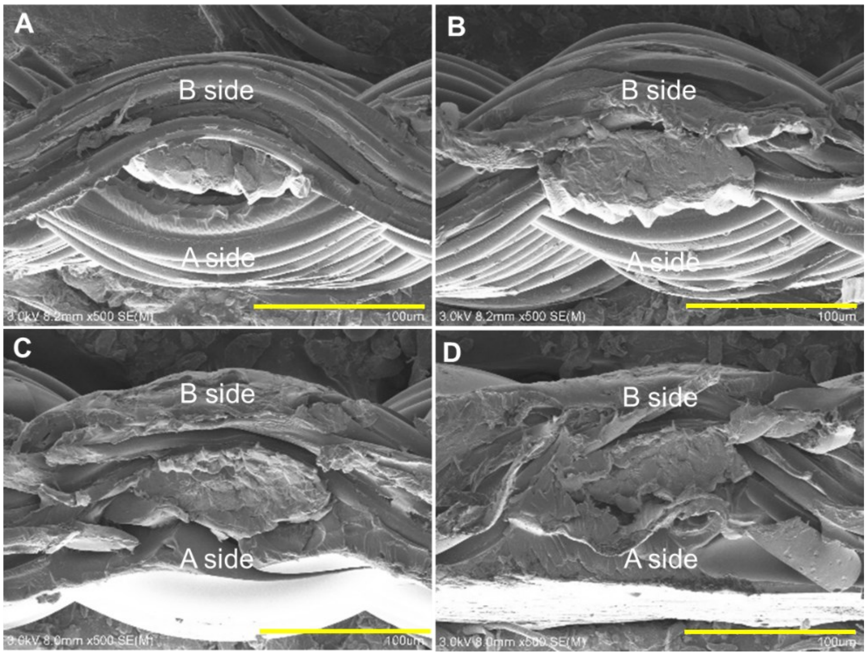


**Supplementary Figure 6.** Cross-sectional SEM images of as-prepared PPFMs (MMA/DEAEMA ratio of 0.5) on two sides. (A) 150 μm, (B) 200 μm, (C) 250 μm, (D) 300 μm. The scale bar is 100 μm.

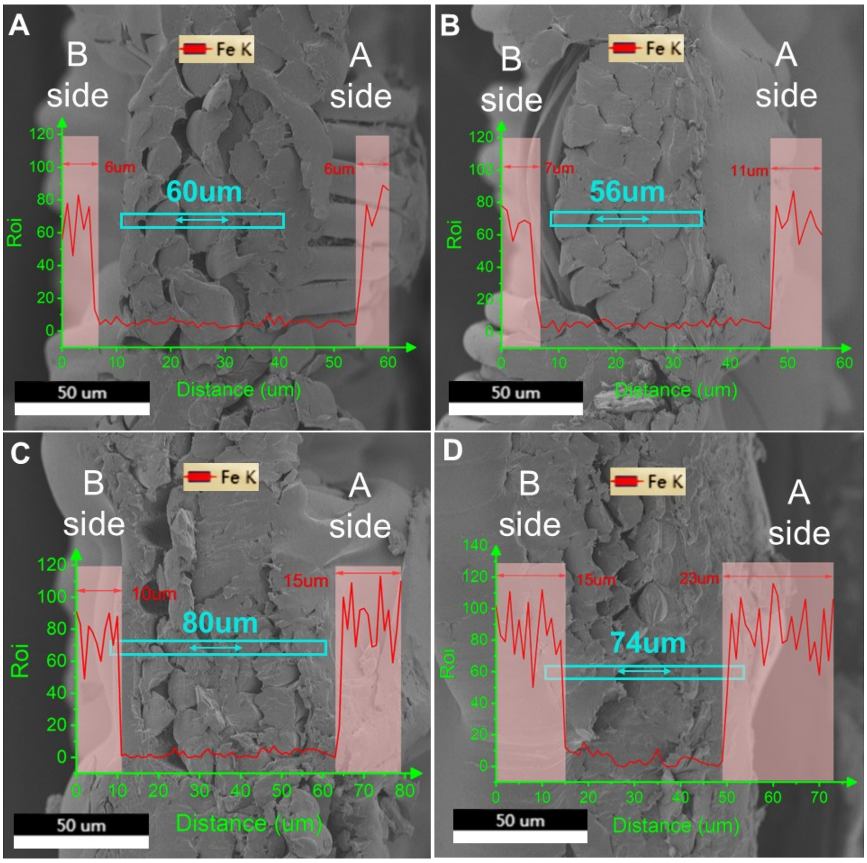


**Supplementary Figure 7.** EDS line-scan element profile of as-prepared PPFMs (MMA/DEAEMA ratio of 0.5) with different gap widths. (A) 150 μm, (B) 200 μm, (C) 250 μm, (D) 300 μm. Fe element is used as tracer agent for measuring the copolymer coating thickness of the membrane on both sides.

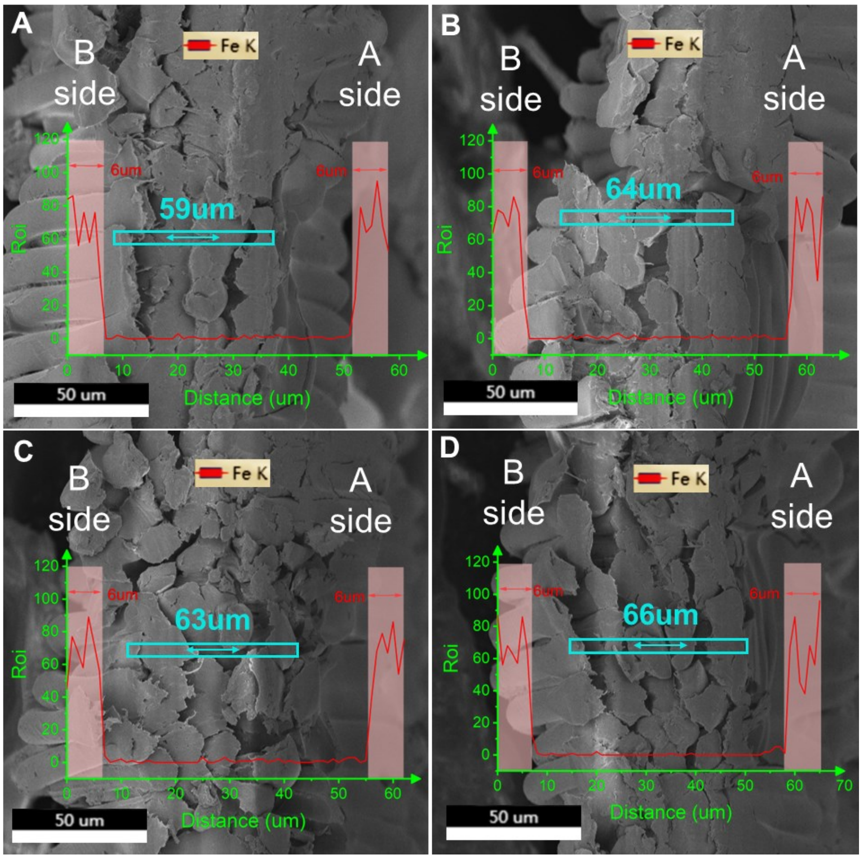


**Supplementary Figure 8.** EDS line-scan element profile of as-prepared PPFMs with different MMA/DEAEMA ratios. (A) 0.5:1, (B) 1:1, (C) 1.5:1 and (D) 2:1. Fe element is used as tracer agent for measuring the copolymer coating thickness of the membrane on both sides. The gap width used is 150 μm.

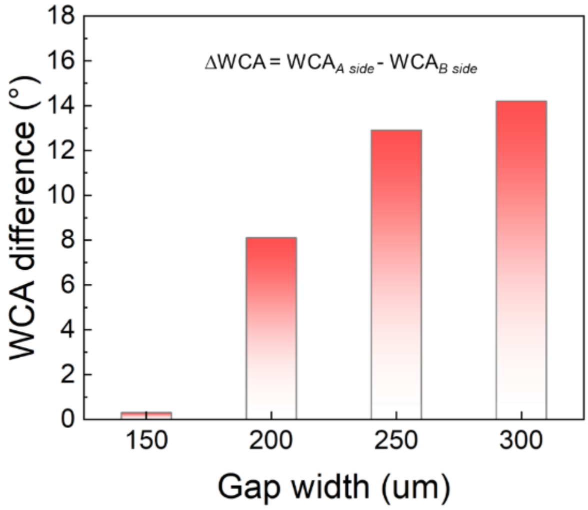


**Supplementary Figure 9.** WCAs difference of as-prepared PPFM surfaces on two sides with different gap width.

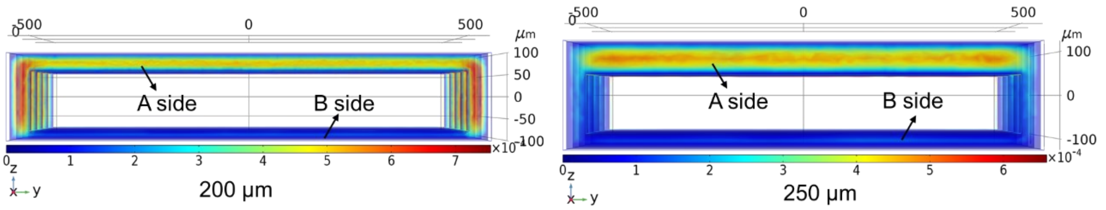


**Supplementary Figure 10.** Velocity field of the copolymer solution on each side under different gap width based on the COMSOL simulation.

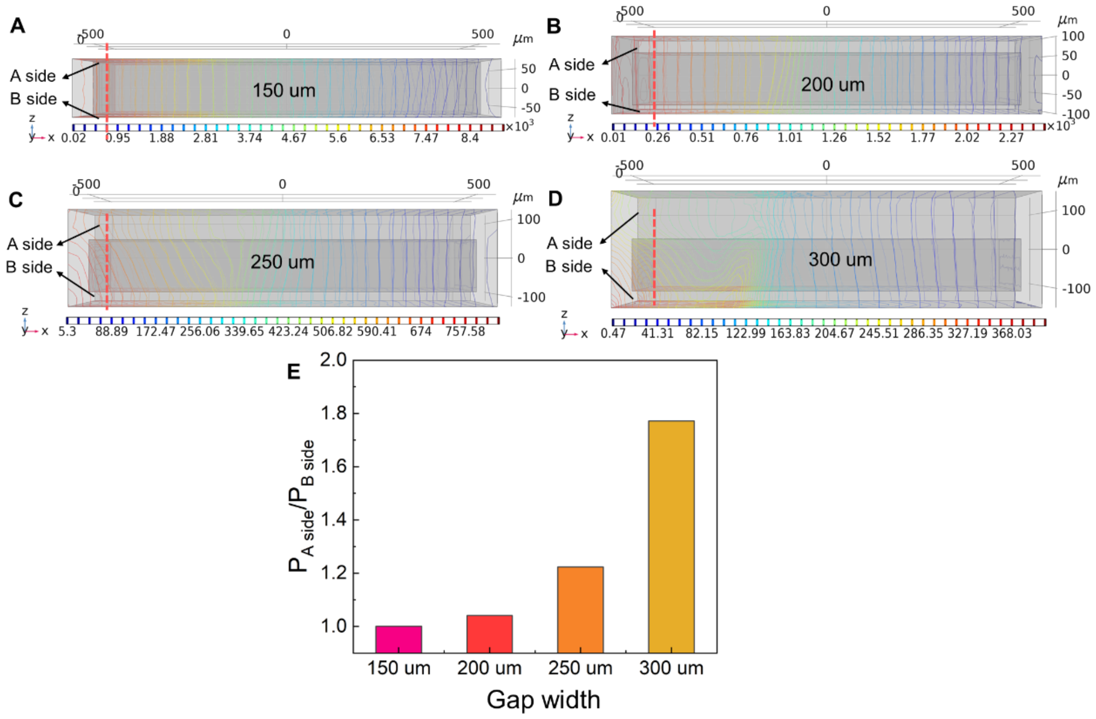


**Supplementary Figure 11.** (A~D) Pressure field of the copolymer solution on each side under different gap width based on the COMSOL simulation. (E) Ratio of pressures between A side and B side calculated by data at red dashed line.

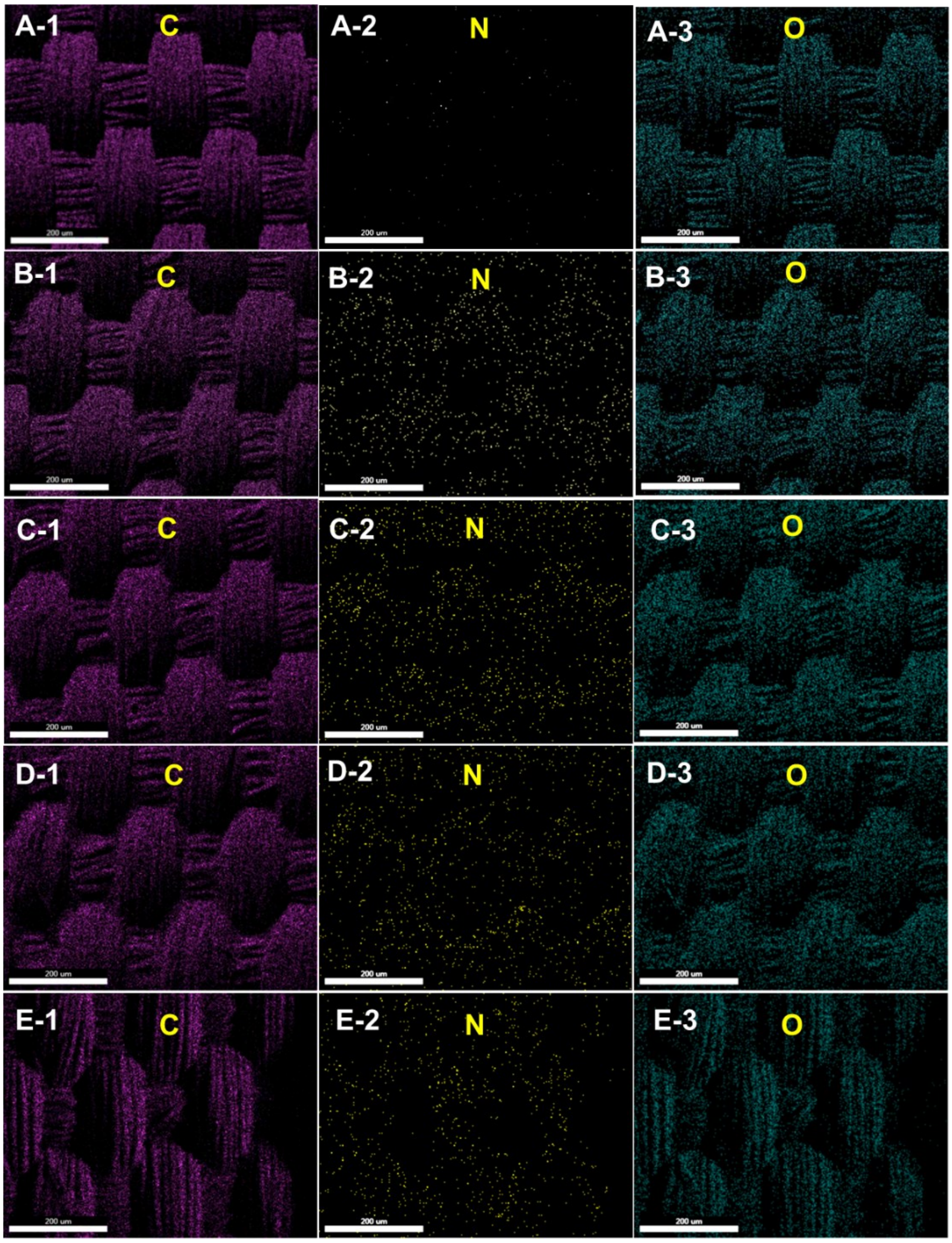


**Supplementary Figure 12.** EDX element mapping of PPFM with different ratios of MMA/DEAEMA. (A) Pristine fabric (B) 0.5:1, (C) 1:1, (D) 1.5:1 and (E) 2:1. The scale bar is 200 μm. The gap width used is 150 μm.

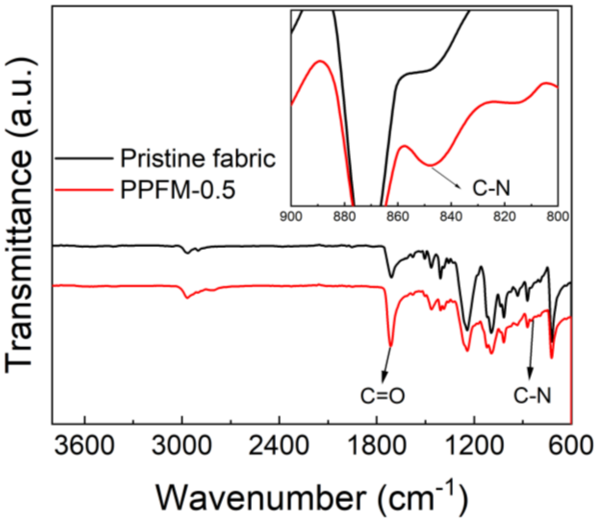


**Supplementary Figure 13**. FT-IR spectra of PPFM-0.5 with gap width of 150 μm and pristine fabric.

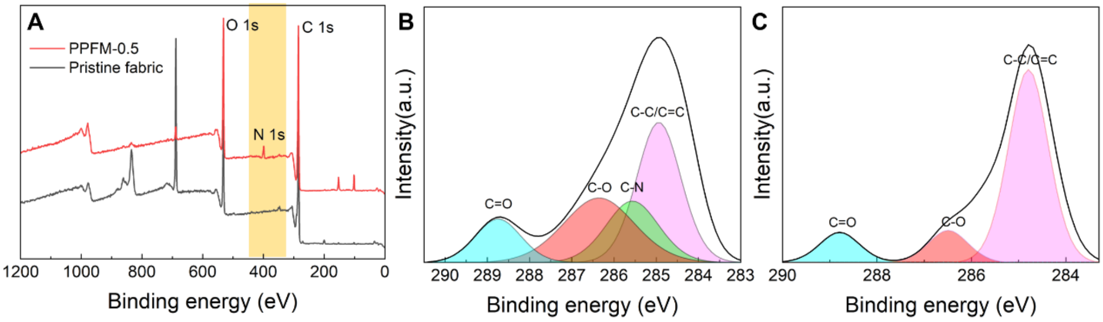


**Supplementary Figure 14.** XPS spectra (A) of PPFM-0.5 with gap width of 150 μm and pristine fabric and the corresponding high-resolution XPS spectra of C 1s of PPFM-0.5 (B) and pristine (C).

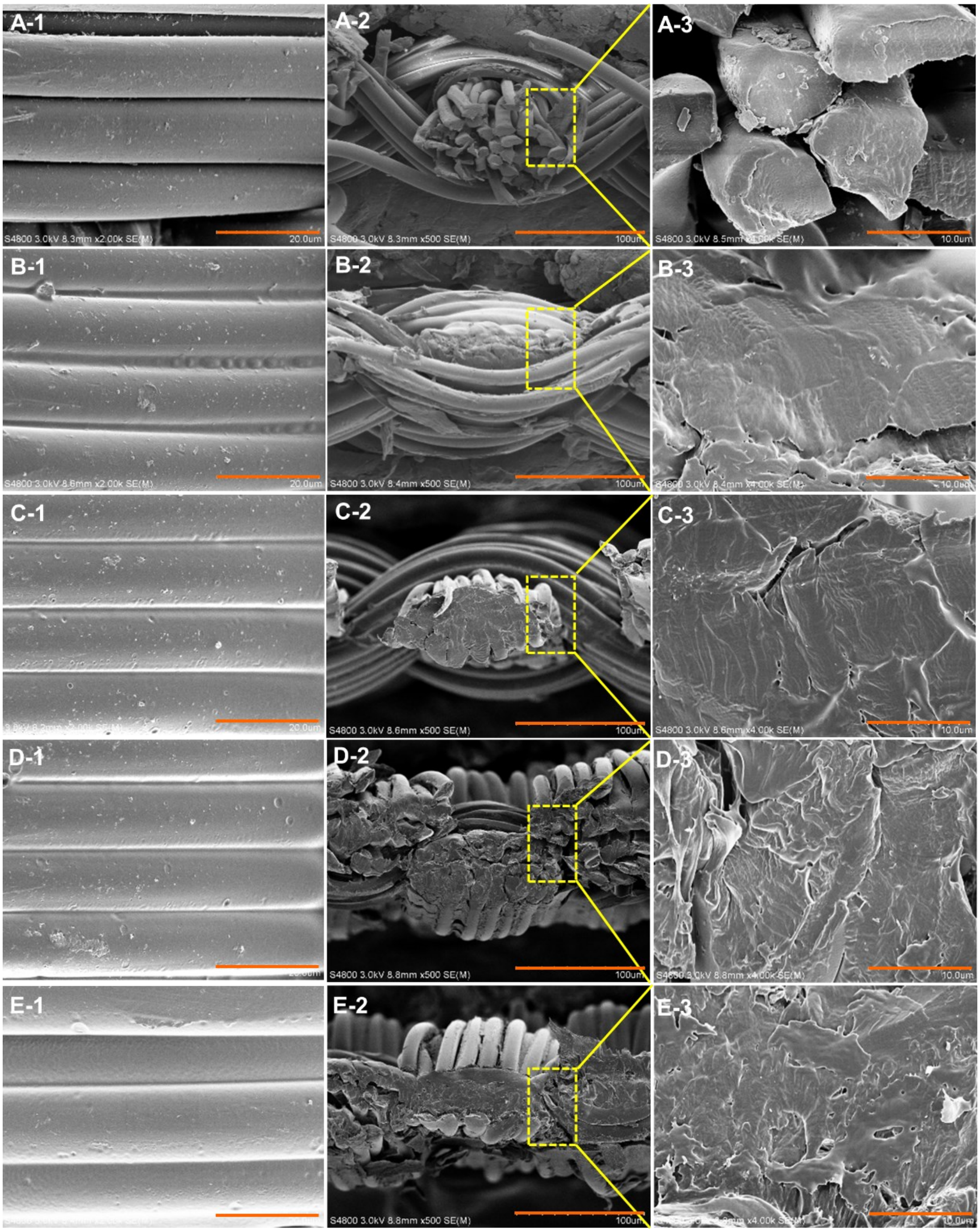


**Supplementary Figure 15.** Surface (A-1~E1) and cross-sectional (A-2~E3) SEM images of PPFM with different ratios of MMA/DEAEMA. (A) Pristine fabric (B) 0.5:1, (C) 1:1, (D) 1.5:1 and (E) 2:1. The scale bar is 20 μm for (A-1~E1), 100 μm for (A-2~E2) and 10 μm for (A-3~E3), respectively. The gap width used is 150 μm.

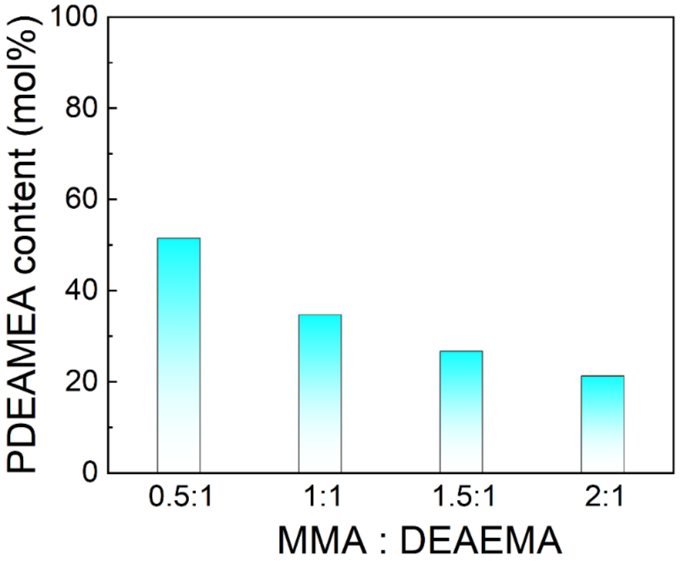


**Supplementary Figure 16.** PDEAEMA content in synthesized PMMA-co-PDEAEMA copolymer under different ratio of MMA/DEAEMA.

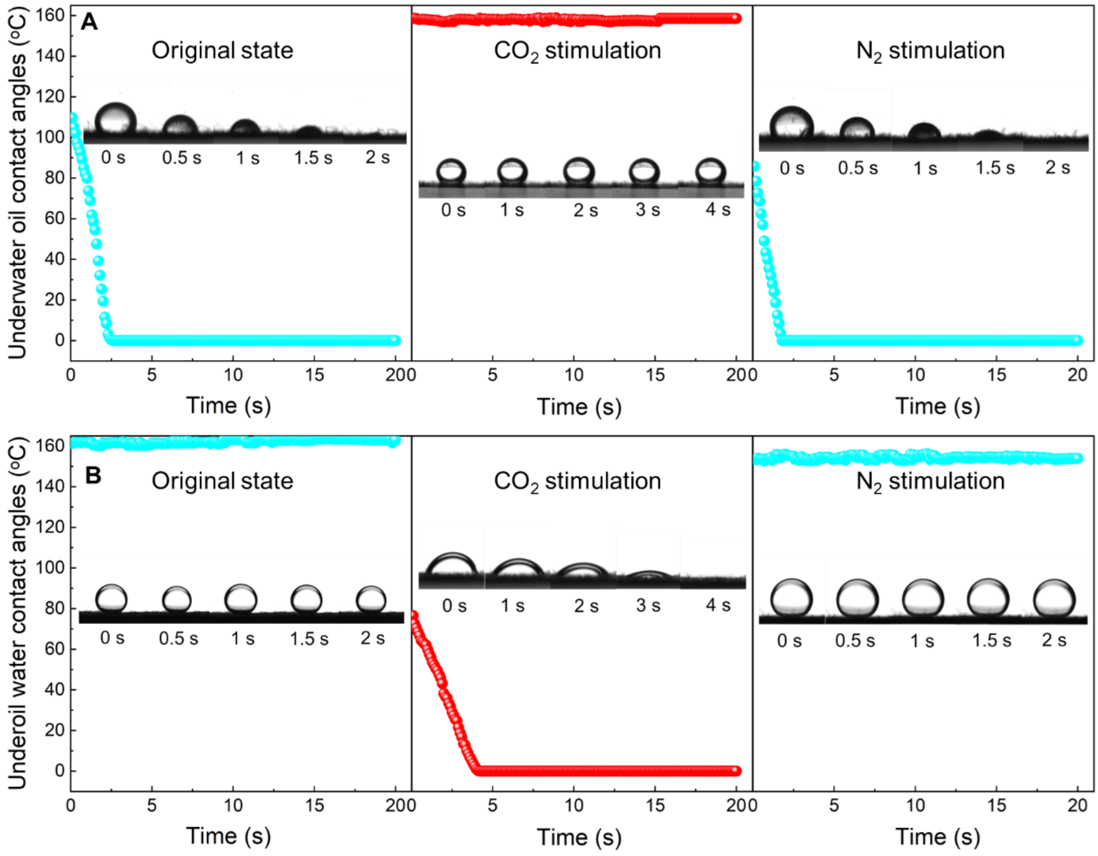


**Supplementary Figure 17.** Underwater oil contact angle (UOCA) (A) and underoil water contact angle (UWCA) (B) of PPFM-0.5 with gap width of 150 μm under alternant $CO_2/N_2$ bubbling.

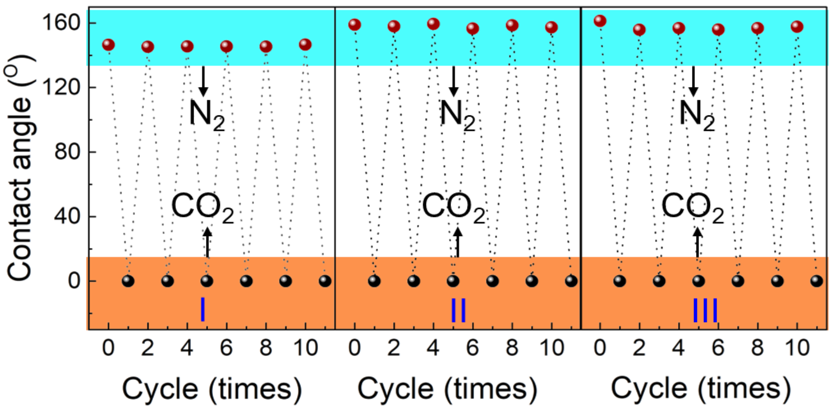


**Supplementary Figure 18.** Contact angle transition of the PPFM-0.5 with a cyclic treatment of $CO_2$ and $N_2$ under different conditions. Contact angles for I, II and III are WCA in air, underwater oil contact angle (UOCA), and underoil water contact angle (UWCA), respectively. The gap width used is 150 μm.

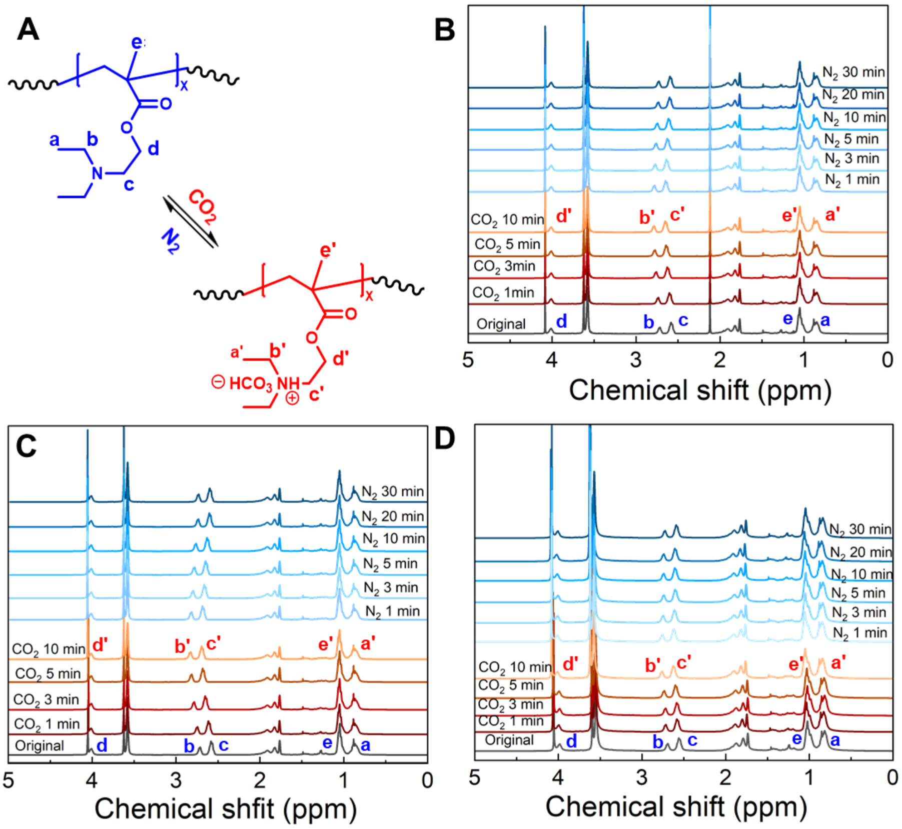


**Supplementary Figure 19.** $^1$H NMR spectra of PMMA-co-PDEAEMA copolymer with different ratio of MMA/DEAEMA in $D_2O$ and tetrahydrofuran-D8 (1:1) before and after bubbling $CO_2$. (A) Reversible protonation and deprotonation reactions of the copolymer upon $CO_2$ or $N_2$ stimulation. (B) ratio of MMA/DEAEMA: 1:1, (C) ratio of MMA/DEAEMA: 1.5:1, (D) ratio of MMA/DEAEMA: 2:1

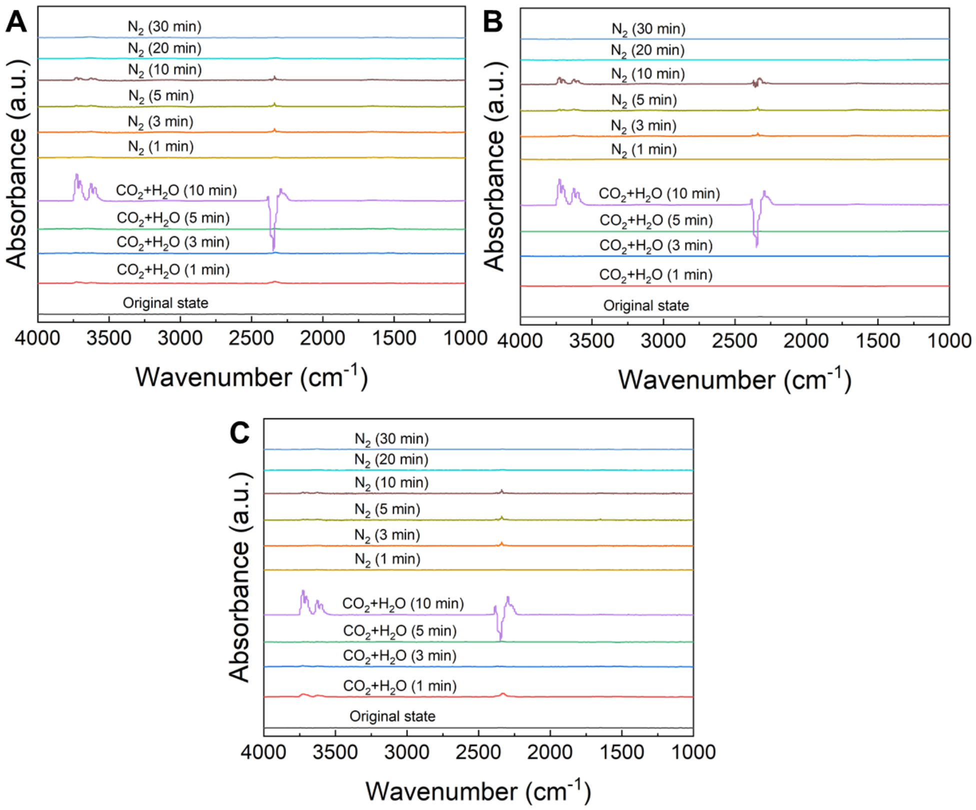


**Supplementary Figure 20.** In-situ FTIR spectra of PPFMs with different ratio of MMA/DEAEMA under the $CO_2$/$N_2$ stimulation. (A) ratio of MMA/DEAEMA: 1:1, (B) ratio of MMA/DEAEMA: 1.5:1, (C) ratio of MMA/DEAEMA: 2:1. The gap width used is 150 μm.

In situ FT-IR measurements were performed using a Nicolet 6700 spectrometer equipped with a reaction cell. The in situ FT-IR measurements were obtained over a range of 4000 to 650 $cm^{-1}$ by averaging 512 scans at a resolution of 4 $cm^{-1}$. First of all, the sample was loaded into the reaction cell for pre-treatment at 70°C and a flow of $N_2$ with a flow rate of 20 mL/min was fed into the in-situ FT-IR cell for 30 min. After removing surface impurities, cool to room temperature to obtain the background. Then a flow of $H_2O$ and $CO_2$ ($H_2O$/$CO_2$=1) mixture with a total flow rate of 20 mL/min was fed into the in-situ FT-IR cell and infrared spectra were collected at the specified time (1, 3, 5 and 10 min). Finally, a flow of $N_2$ was fed into the in-situ FT-IR cell at a flow rate of 20 mL/min, and infrared spectra were collected at the specified time (1, 3, 5, 10, 20 and 30 min).

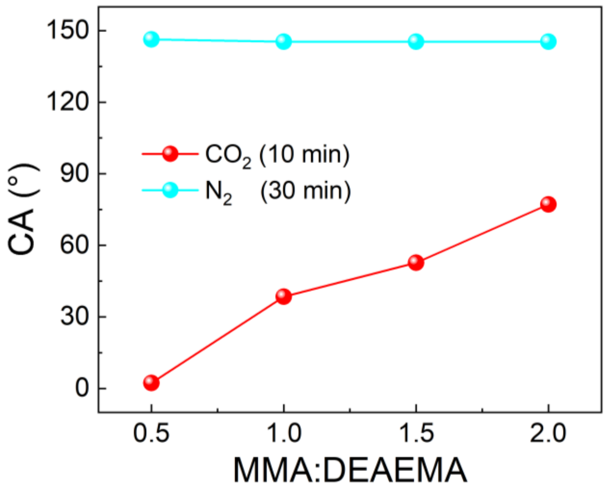


**Supplementary Figure 21.** Wettability difference of the PPFMs surface with different ratio of MMA/DEAEMA. The gap width used is 150 μm.

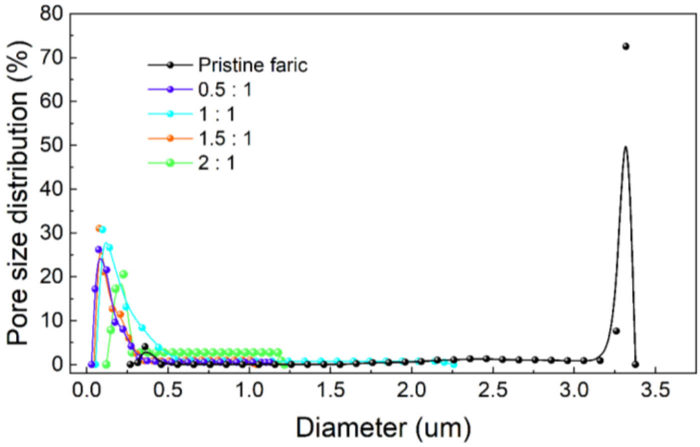


**Supplementary Figure 22.** Pore size distribution of the PPFMs with different ratio of MMA/DEAEMA. The gap width used is 150 μm.

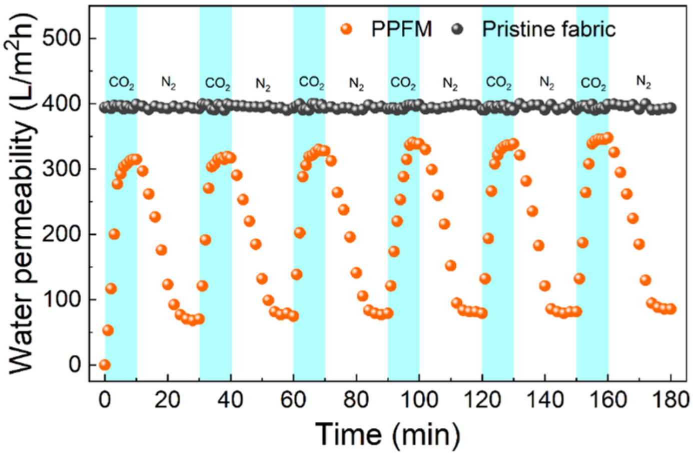


**Supplementary Figure 23.** Reversible water permeability decreases and recovers upon $CO_2$ and

$N_2$ bubbling, respectively, and the results of pristine fabric and PPFM-0.5 with gap width of 150 μm are shown for comparison.

To verify the effective gas-responsive gating function of PPFM, water filtration was performed at 25 °C under $CO_2/N_2$ stimulation, using a vacuum filtration system under 10 kPa. As shown in Supplementary Fig. 24, dead-end filtration cell with effective membrane area of 3.14 $cm^2$ was used. For $CO_2$ induced switching studies, the membrane was filtered with DI water first for a period of time to ensure a steady state. Then firstly bubbling $CO_2$ into solution for 10 min, next bubbling $N_2$ for 20 min. Water flux samples were taken every 1 min for $CO_2$ and 2 min for $N_2$. The flux was calculated from the volume of solution permeated per unit time and per unit area of the membrane surface according to equation (S8).

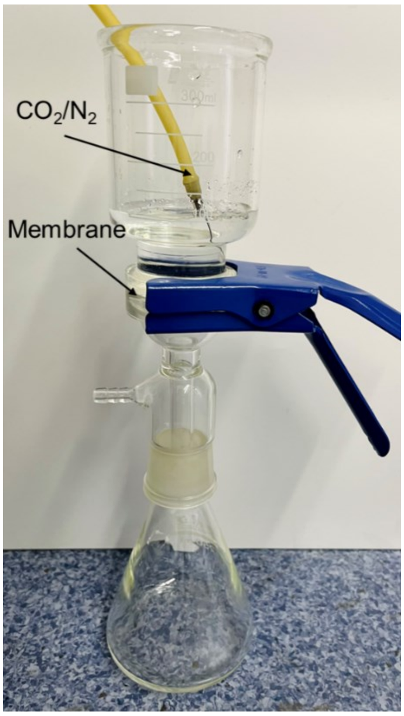


**Supplementary Figure 24.** Experimental setup used for gas-responsive water flux measurements

Supplementary Table 2. Variation of the oil and water intrusion pressure of PPFM-0.5 with gap width of 150 μm under $CO_2$/$N_2$ stimulation

| | Water | n-Hexane | Toluene | Isooctane |
|---|---|---|---|---|
| Dried state[a] | 17 kPa | 0 kPa | 0 kPa | 0 kPa |
| In presence of $N_2$[b] | 6 kPa | 0 kPa | 0 kPa | 0 kPa |
| In presence of $CO_2$[c] | 0 kPa | 24 kPa | 22 kPa | 18 kPa |

[a] The dried state is realized by continuously purging surface with $N_2$ for a long time. [b] $N_2$ bubbling time is 20 min. [c]$CO_2$ bubbling time is 10 min.

As shown in the Supplementary Table 2, all the oil intrusion pressures of PPFM-0.5 in dried state are 0 kPa while the water intrusion pressure is 17 kPa, which is due to hydrophobicity of membrane surface. After treated with $N_2$ bubbling for 20 min, the oil intrusion pressures of PPFM-0.5 remain unchanged while the water intrusion pressure decreases from 17 kPa to 6 kPa. This is mainly because $CO_2$ is not completely removed from water in this case, keeping some hydrophilic and protonated groups still existing in the membrane.

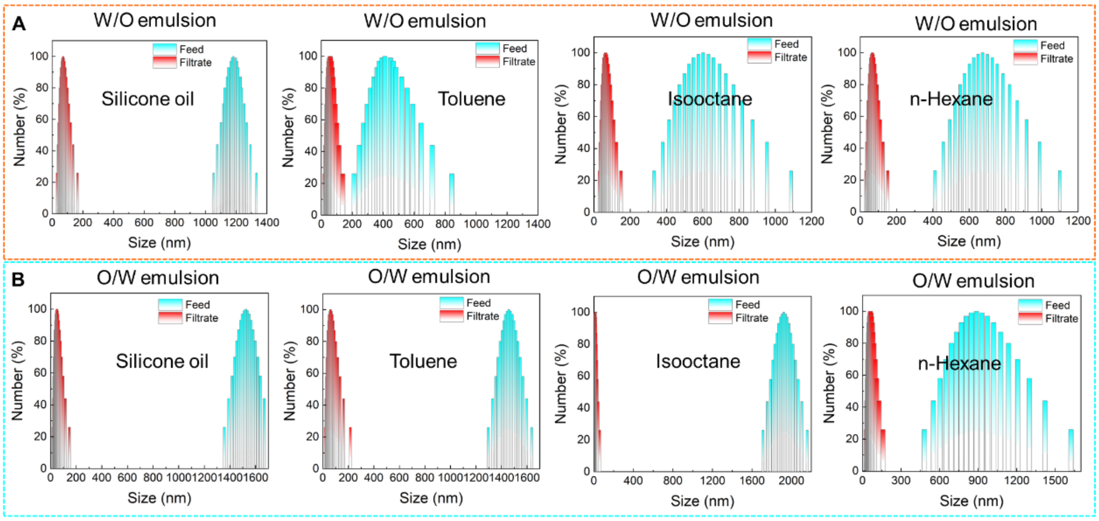


**Supplementary Figure 25.** Variation of droplet size between the feed and the filtrate under $CO_2$/$N_2$ stimulation. (A): W/O emulsion, (B): O/W emulsion

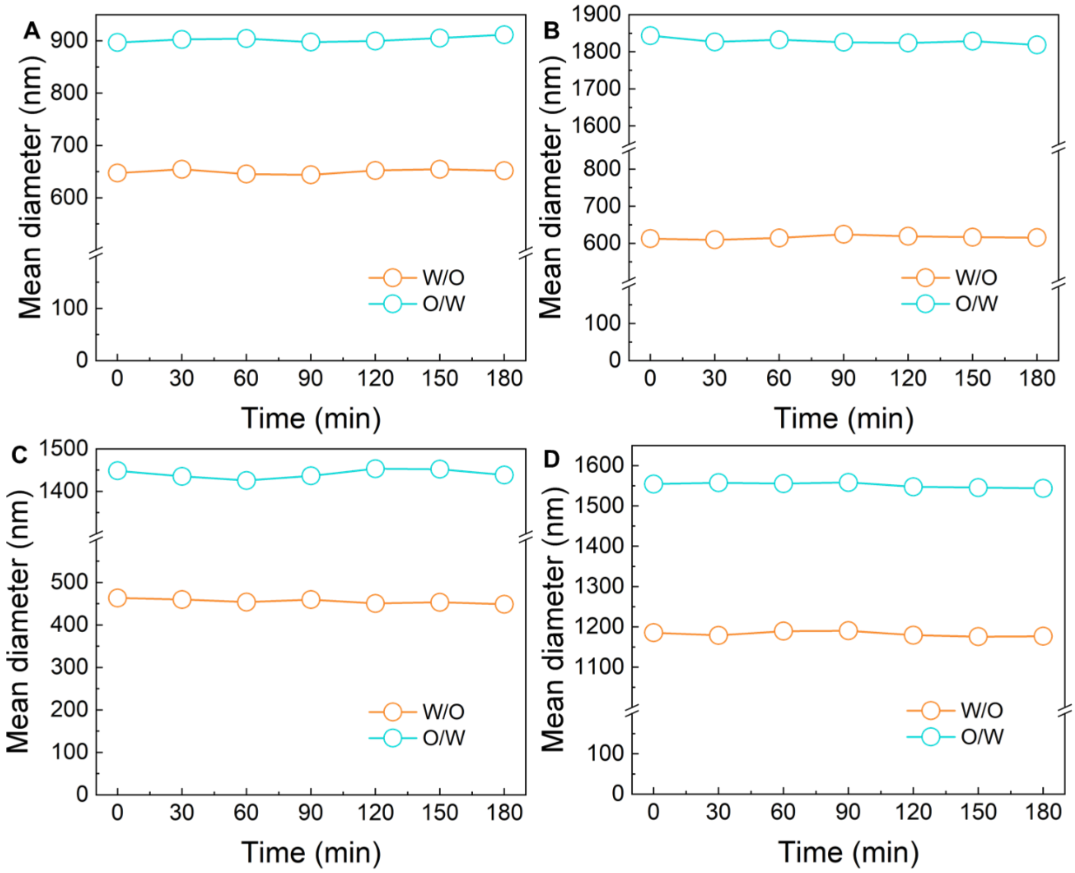


**Supplementary Figure 26.** Variation of droplet sizes of various O/W and W/O emulsions under $CO_2$ stimulation. (A) n-Hexane, (B) Isooctane, (C) Toluene, (D) silicone oil.

The stability of emulsion under $CO_2$ stimulation is of importance to emulsion separation. As shown in Supplementary Fig. 26, after bubbling $CO_2$ into O/W and W/O emulsions for 3h, there are no changes in the mean diameters of all emulsions in the whole process, confirming that $CO_2$ bubbling has little effect on the stability of emulsions.

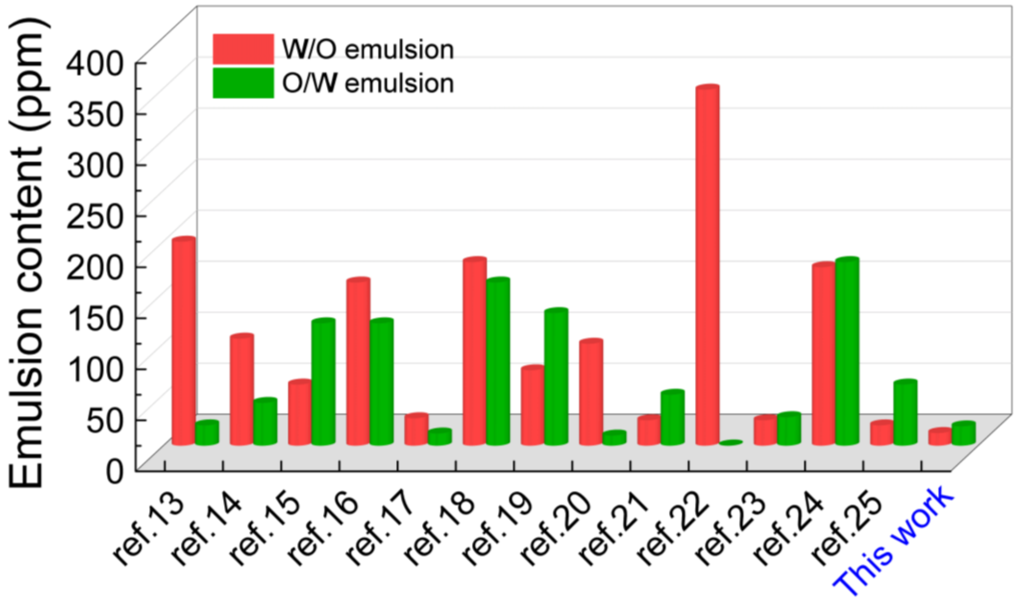


**Supplementary Figure 27.** Comparison of the emulsion content in the filtrates of the PPFM-0.5 with the state-of-art membranes reported in the literature[13-25]. The oil in both W/O and O/W emulsion is n-hexane. The gap width used is 150 μm.

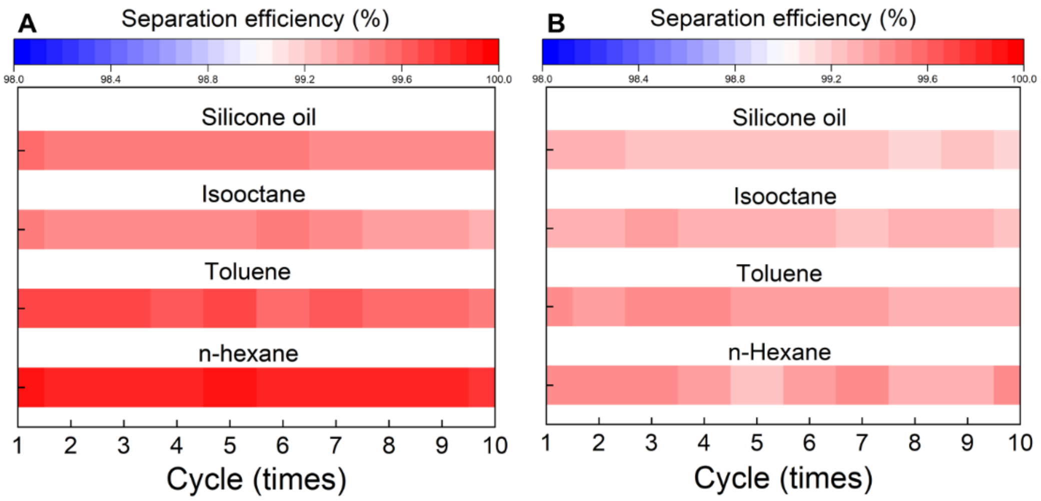


**Supplementary Figure 28.** Recyclability of the PPFM-0.5 with gap width of 150 μm for various types of W/O emulsions (A) and O/W emulsions (B).

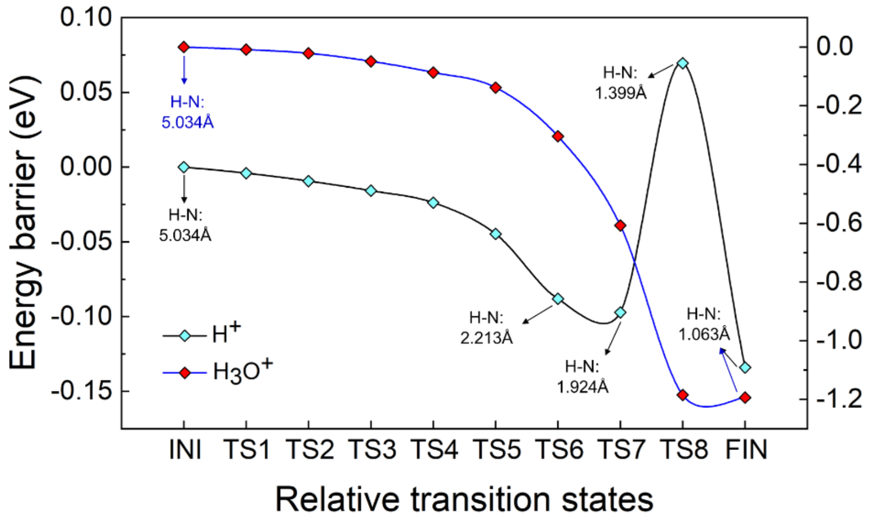


**Supplementary Figure 29.** Energy barrier diagram of different protonation paths on DEAEMA. Black and blue lines represent the protonation pathway of $H^+$ and $H_3O^+$, respectively.

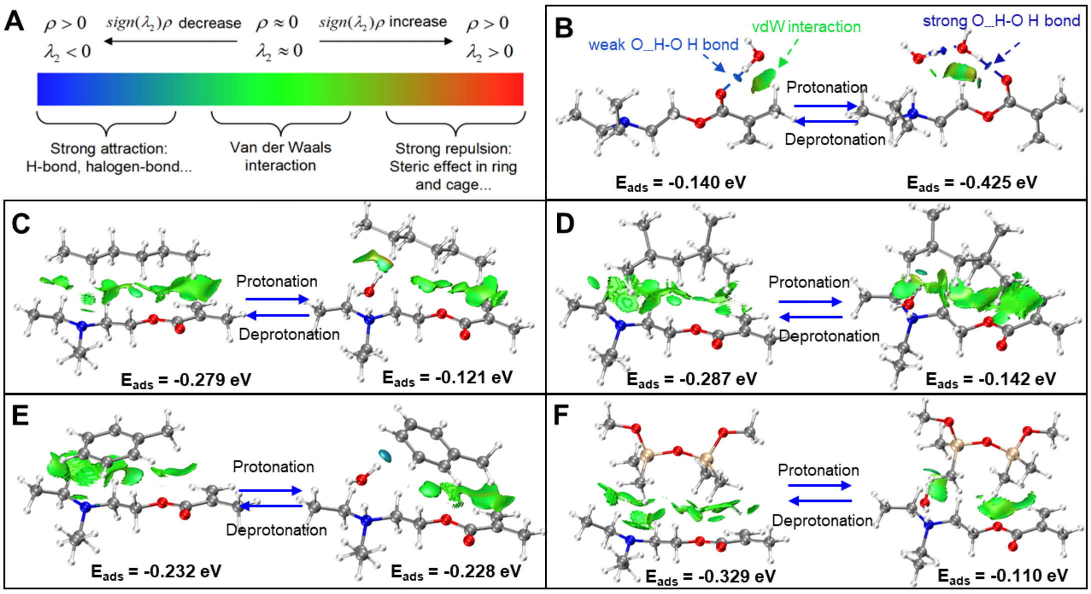


**Supplementary Figure 30.** (A) Common interpretation of coloring method of mapped function sign($\lambda_2$)$\rho$ in IGMH map. (B-F) The spatial isosurface diagrams of $\delta g^{inter}$ for substrates and adsorbed species. The coloring method of sign($\lambda_2$)$\rho$ is the same as shown above. In the IGMH calculation each oil molecule is defined as one segment and the others as another segment. Grey, white, red, blue and brown balls represent the C, H, O, N and Si atoms, respectively.

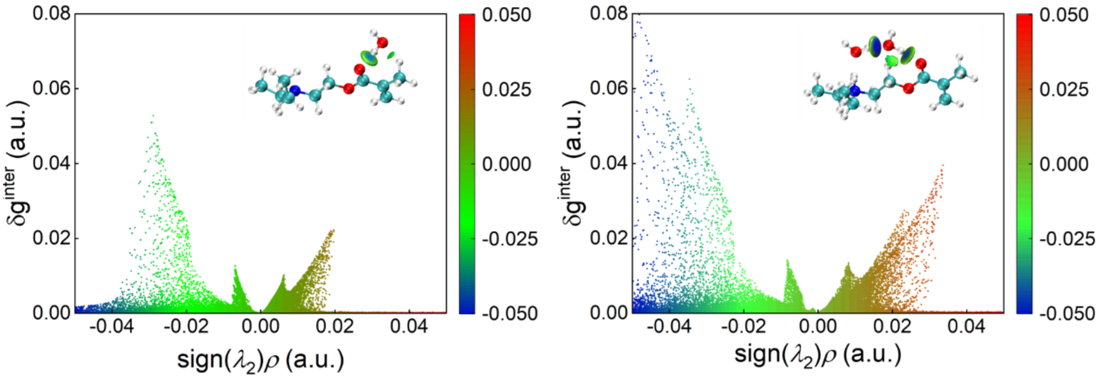


**Supplementary Figure 31.** 2D scatter plot of non-covalent interactions between substrate and adsorbed $H_2O$ (right) before the protonation of DEAEMA and (left) after the protonation of DEAEMA

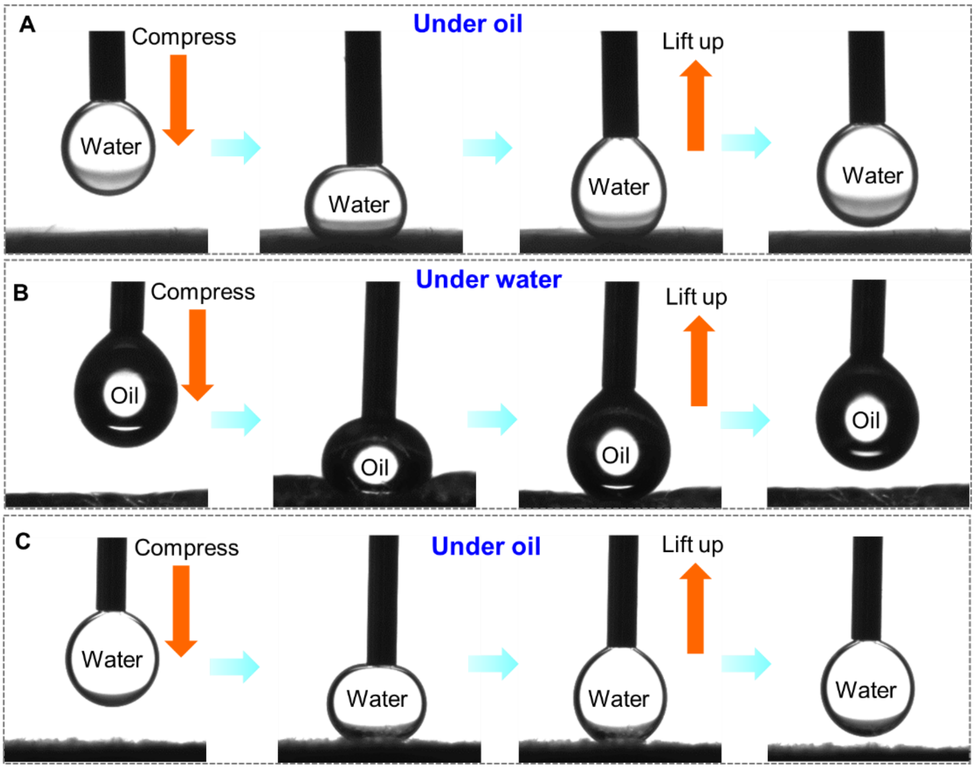


**Supplementary Figure 32.** Photographs of the dynamic under-oil water-adhesion (A, C) and under-water oil-adhesion (B) measurement on the PPFM-0.5 surface with gap width of 150 μm.

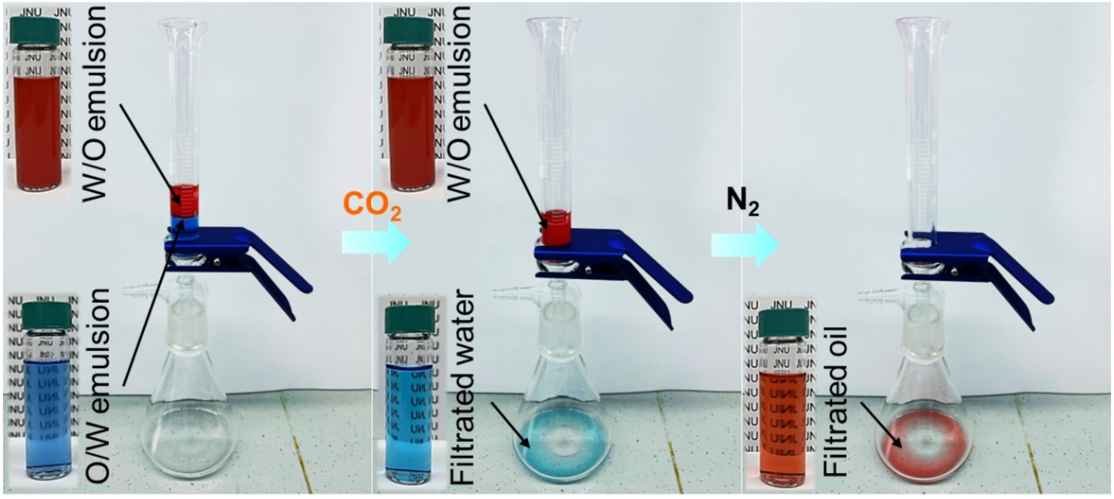


**Supplementary Figure 33.** Two-step separation process for multiphase emulsions system under $CO_2/N_2$ stimulation.

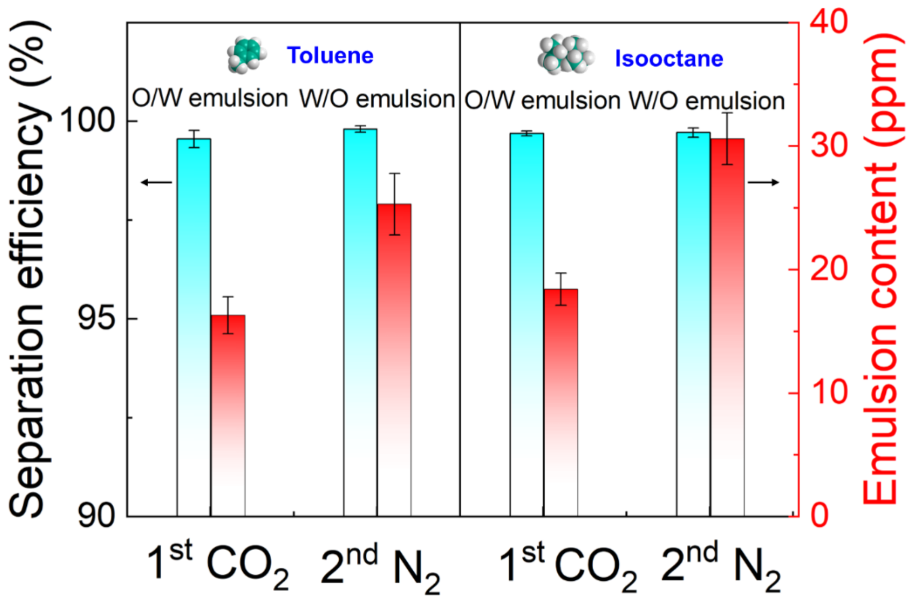


**Supplementary Figure 34.** Emulsion content in filtrates of PPFM-0.5 with gap width of 150 μm in 1st and 2nd step operation. The emulsion content is oil content for O/W emulsion and water content for W/O emulsion, respectively. The error bars represent the standard deviation and were calculated on the basis of at least three data points measured from different samples.

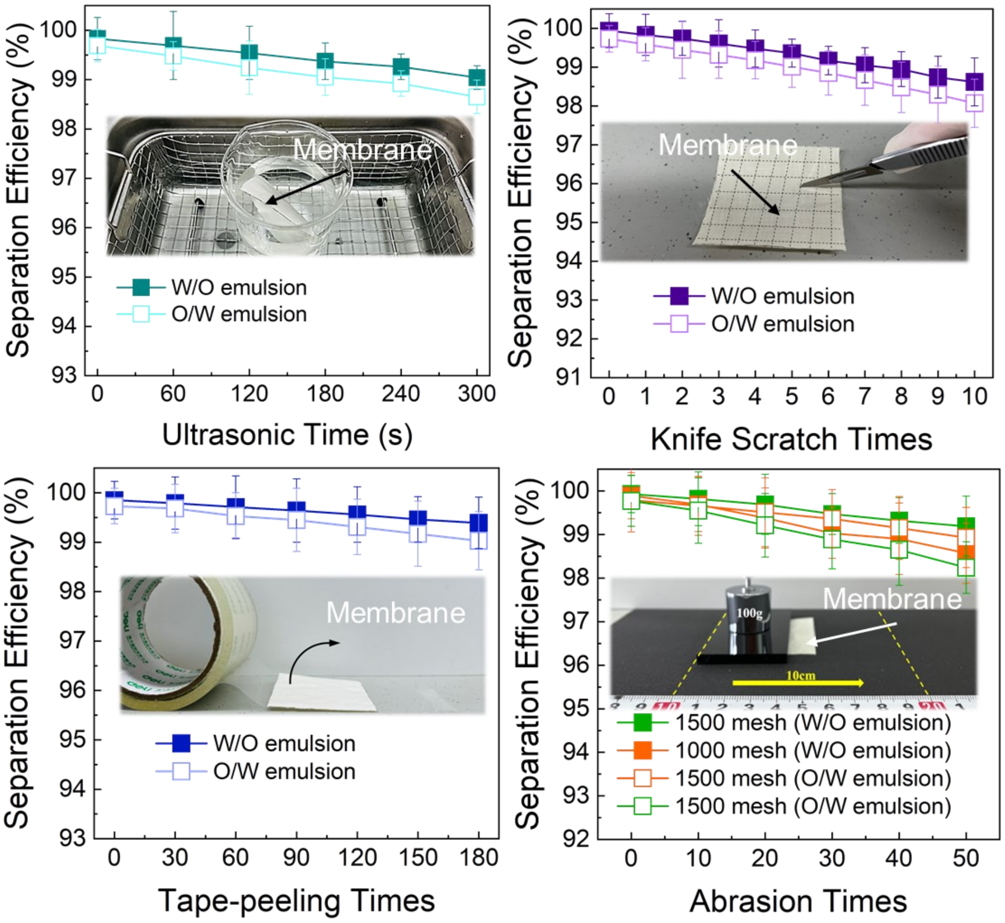


**Supplementary Figure 35.** Separation efficiency of PPFM-0.5 with gap width of 150 μm toward W/O and O/W emulsion under multi-cycling mechanical tests. The W/O and O/W emulsion used is water/*n*-hexane and *n*-hexane/water emulsion, respectively. The error bars represent the standard deviation and were calculated on the basis of at least three data points measured from different samples.

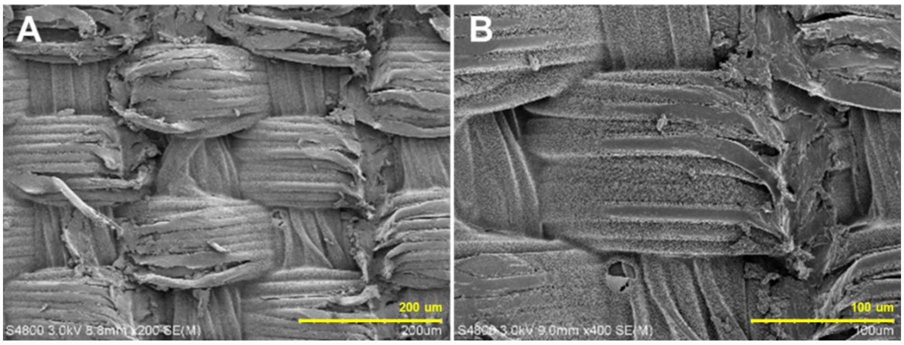


**Supplementary Figure 36.** Surface SEM image of the PPFM-0.5 with gap width of 150 μm before (A) and after ultrasonic time of 300 s (B).

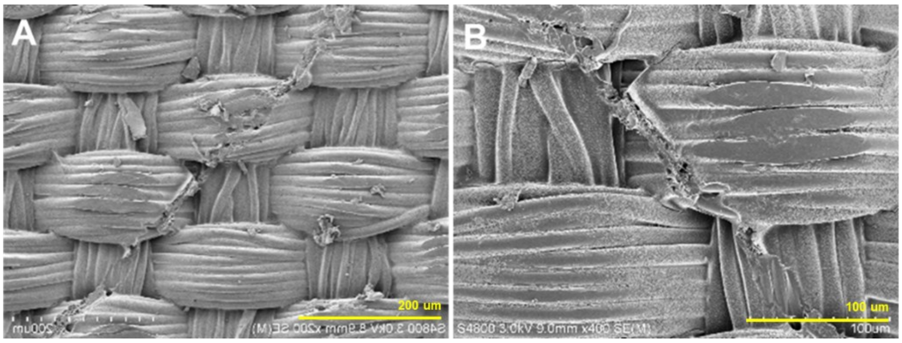


**Supplementary Figure 37.** Surface SEM image of the PPFM-0.5 with gap width of 150 μm before (A) and after scratch times of 10 (B).

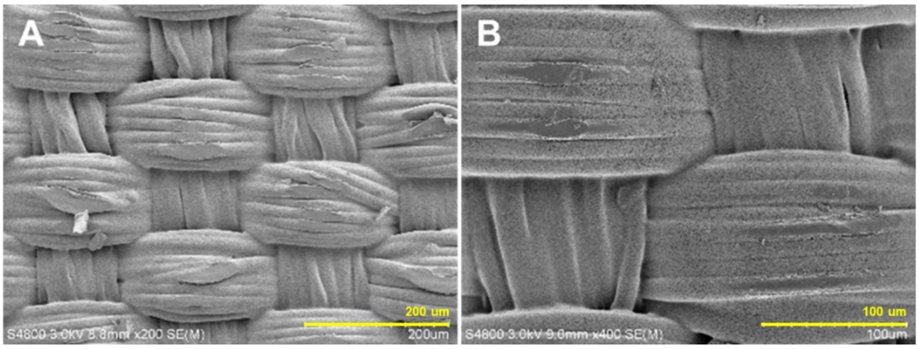


**Supplementary Figure 38.** Surface SEM image of the PPFM-0.5 with gap width of 150 μm before (A) and after tape-peeling times of 180 (B).

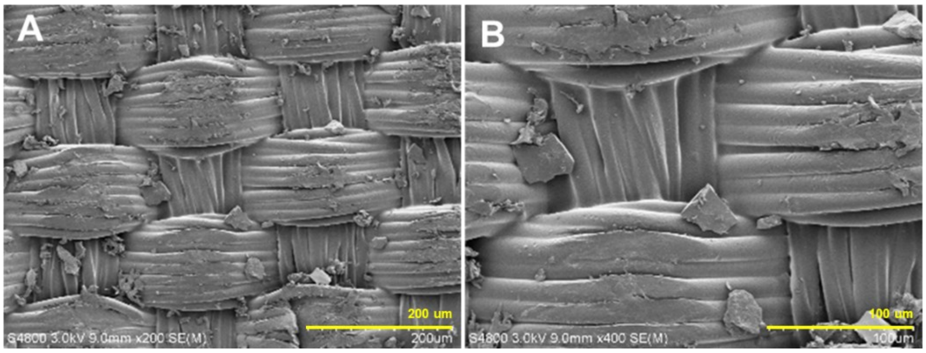


**Supplementary Figure 39.** Surface SEM image of the PPFM-0.5 with gap width of 150 μm before (A) and after abrasion times of 50 (B).

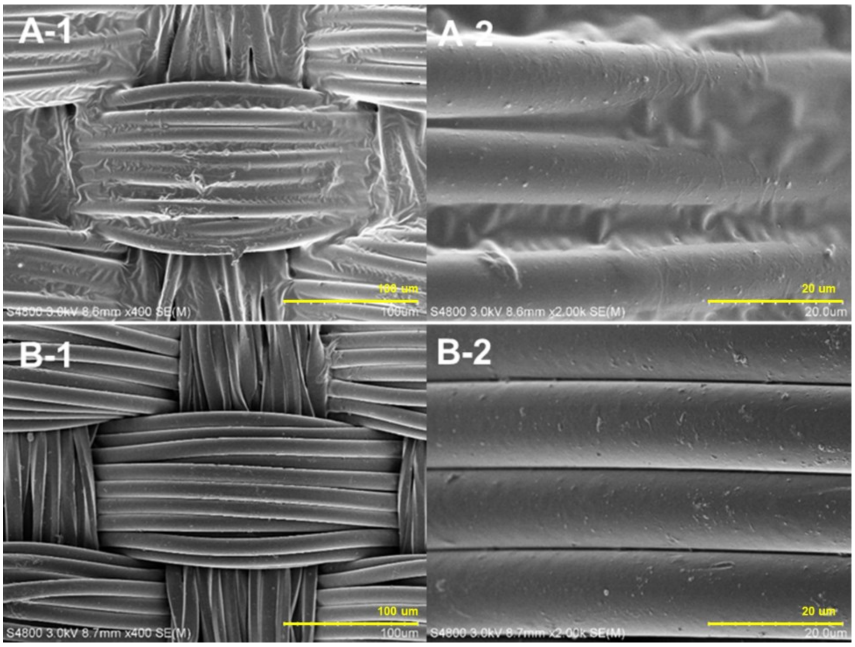


**Supplementary Figure 40.** Surface SEM image of the PPFM-0.5 with gap width of 150 μm after water/*n*-hexane emulsion separation (A) and cleaned by $CO_2$ (B).

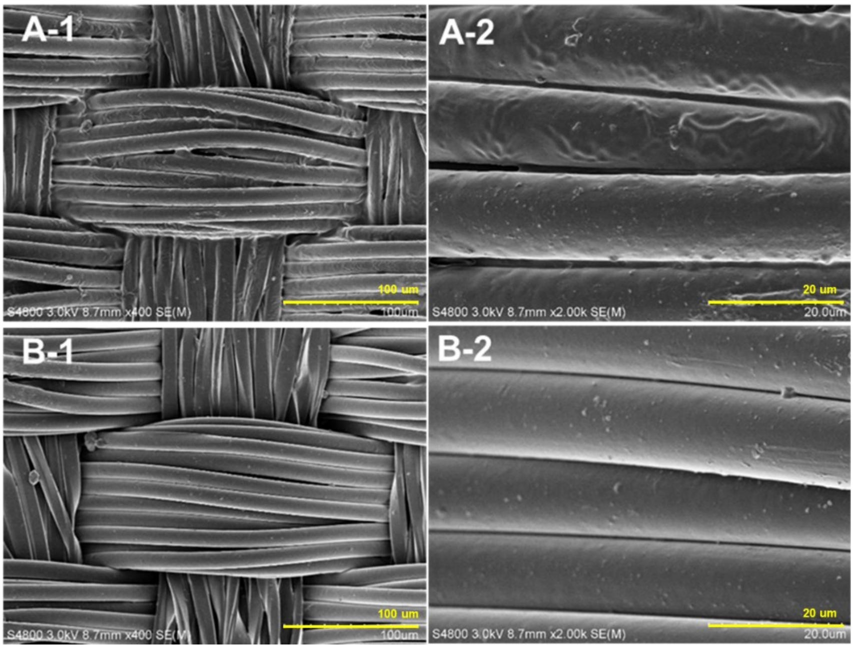


**Supplementary Figure 41.** Surface SEM image of the PPFM-0.5 with gap width of 150 μm after *n*-hexane/water emulsion separation (A) and cleaned by $CO_2$ (B).

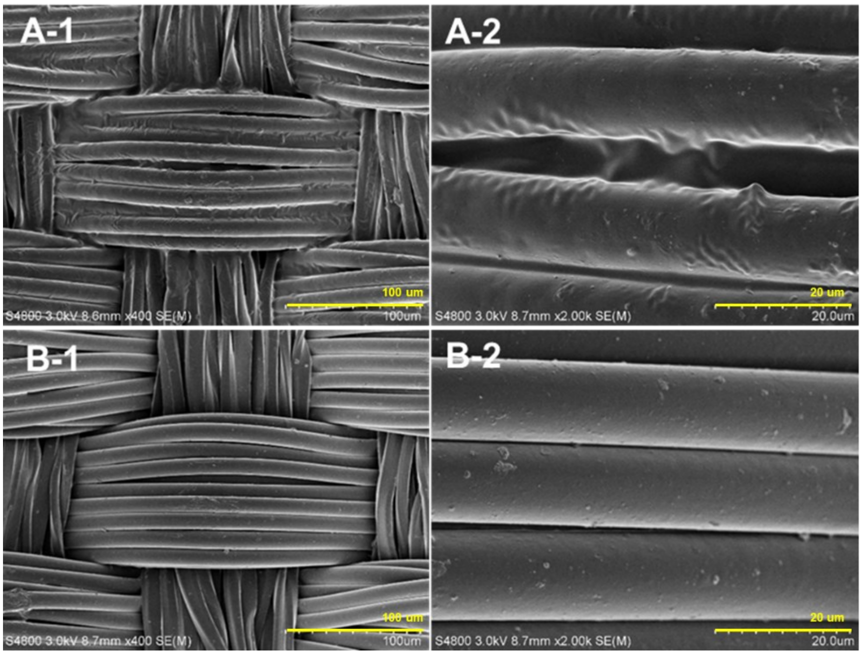


**Supplementary Figure 42.** Surface SEM image of the PPFM-0.5 with gap width of 150 μm after *n*-hexane/water emulsion containing TC separation (A) and cleaned by $CO_2$ (B).

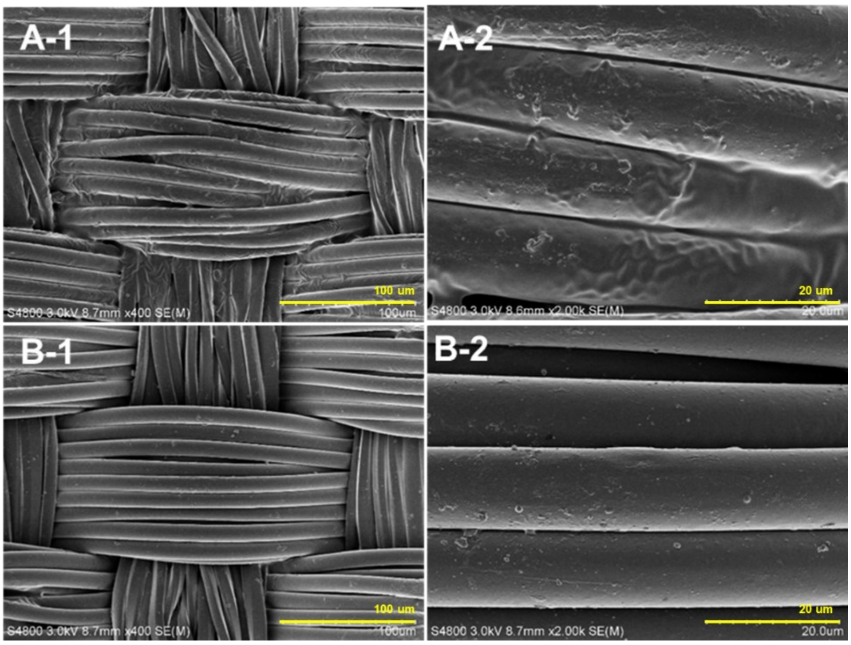


**Supplementary Figure 43.** Surface SEM image of the PPFM-0.5 with gap width of 150 μm after *n*-hexane/water emulsion containing BSA separation (A) and cleaned by $CO_2$ (B).

## Supplementary References:


1. S. Arias, A. Montlaur. Numerical study and experimental comparison of two-phase flow generation in a T-junction. *AIAA J.* **55**, 1565-1574 (2017).

2. W. Lan, S. Li, Y. Wang, G. Luo. CFD simulation of droplet formation in microchannels by a modified level set method. *Ind. Eng. Chem. Res.* **53**, 4913-4921 (2014).
3. G. Kresse, J. Furthmüller. Efficient iterative schemes for ab initio total-energy calculations using a plane-wave basis set. *Phys. Rev. B* **54**, 11169 (1996).
4. J. P. Perdew, K. Burke, M. Ernzerhof. Generalized gradient approximation made Simple. *Phys. Rev. Lett.* **77**, 3865 (1996).
5. S. Grimme, J. Antony, S. Ehrlich, H. Krieg. A consistent and accurate ab initio parametrization of density functional dispersion correction (DFT-D) for the 94 elements H-Pu. *J. Chem. Phys.* **132**, 154104 (2010).
6. G. Kresse, D. Joubert. From ultrasoft pseudopotentials to the projector augmented-wave method. *Phys. Rev. B* **59**, 1758 (1999).
7. H. J. Monkhorst, J. D. Pack. Special points for brillouin-zone integrations. *Phys. Rev. B* **13**, 5188-5192(1976).
8. K. Mathew, R. Sundararaman, K. Letchworth-Weaver, T. A. Arias, R. G. Hennig. Implicit solvation model for density-functional study of nanocrystal surfaces and reaction pathways. *J. Chem. Phys.* **140**, 084106 (2014).
9. T. Lu, F. Chen. Multiwfn: a multifunctional wavefunction analyzer. *J. Comput. Chem.* **33**, 580-592 (2012).
10. T. Lu, Q. Chen. Independent gradient model based on hirshfeld partition: a new method for visual study of interactions in chemical systems. *J. Comput. Chem.* **43**, 539-555 (2022).
11. W. Humphrey, A. Dalke, K. Schulten. VMD: visual molecular dynamics. *J. Mol. Graph.* **14**, 33-38 (1996).
12. J. Mo, J. Sha, D. Li, Z. Li, Y. Chen. Fluid release pressure for nanochannels: the Young-Laplace equation using the effective contact angle. *Nanoscale* **11**, 8408-8415 (2019).
13. W. Zhang, et al. Thermo-driven controllable emulsion separation by a polymer-decorated membrane with switchable wettability. *Angew. Chem., Int. Ed.* **57**, 5740 (2018).
14. Y. Qin, et al. Mechanically robust janus poly(lactic acid) hybrid fibrous membranes toward highly efficient switchable separation of surfactant-stabilized oil/water emulsions. *ACS Appl. Mater. Inter.* **12**, 50879-50888 (2020).
15. K. Wang, et al. Multifunctional switchable nanocoated membranes for efficient

integrated purification of oil/water emulsions. *ACS Appl. Mater. Inter.* **13**, 54315-54323 (2021).

16. Y. Long, Y. Shen, H. Tian, Y. Yang, H. Feng, J. Li. Superwettable coprinus comatus coated membranes used toward the controllable separation of emulsified oil/water mixtures. *J. Membrane Sci.* **565**, 85-94 (2018).
17. J. Li, et al. Smart candle soot coated membranes for on-demand immiscible oil/water mixture and emulsion switchable separation. *Nanoscale* **9**, 13610-13617 (2017).
18. C. Yang, et al. Design of a janus F-$TiO_2$@PPS porous membrane with asymmetric wettability for switchable oil/water separation. *ACS Appl. Mater. Inter.* **11**, 22408-22418 (2019).
19. W. Liu, et al. Waste cigarette filter as nanofibrous membranes for on-demand immiscible oil/water mixtures and emulsions separation. *J. Colloid Interf. Sci.* **549**, 114-122 (2019).
20. Y. Lin, et al. Development of janus membrane with controllable asymmetric wettability for highly-efficient oil/water emulsions separation. *J. Membrane Sci.* **606**, (2020).
21. L. Li, et al. Bio-inspired membrane with adaptable wettability for smart oil/water separation. *J. Membrane Sci.* **598**, (2020).
22. H. N. Doan, et al. Environmentally friendly chitosan-modified polycaprolactone nanofiber/nanonet membrane for controllable oil/water separation. *ACS Appl. Polym. Mater.* **3**, 3891-3901 (2021).
23. Y. Kang, et al. PVDF-modified $TiO_2$ nanowires membrane with underliquid dual superlyophobic property for switchable separation of oil-water emulsions. *ACS Appl. Mater. Inter.* **12**, 40925-40936 (2020).
24. R. Qu, et al. Photothermally induced in situ double emulsion separation by a carbon nanotube/poly(N-isopropylacrylamide) modified membrane with superwetting properties. *J. Mater. Chem. A* **8**, 7677-7686 (2020).
25. M. Wu, P. Mu, B. Li, Q. Wang, Y. Yang, J. Li. Pine powders-coated PVDF multifunctional membrane for highly efficient switchable oil/water emulsions separation and dyes adsorption. *Sep. Purif. Technol.* **248**, 117028 (2020).
